# Juvenile dermatomyositis‐associated calcinosis successfully treated with combined immunosuppressive, bisphosphonate, oral baricitinib and physical therapy

**DOI:** 10.1111/dth.15960

**Published:** 2022-11-04

**Authors:** Manuel Agud‐Dios, Jorge Arroyo‐Andres, Carmen Rubio‐Muñiz, Carlos Zarco‐Olivo, Alba Calleja‐Algarra, Jaime de Inocencio, Sara Isabel Palencia Perez

**Affiliations:** ^1^ Department of Dermatology Hospital Universitario 12 de Octubre Madrid Spain; ^2^ Department of Pediatric Rheumatology Hospital Universitario 12 de Octubre Madrid Spain; ^3^ Department of Public Health & Maternal and Child Health Complutense University of Madrid Madrid Spain

Dear Editor,

A 5‐year‐old Romanian boy presented with heliotrope erythema, periungual telangiectasia, Gottron's papules, discrete calcium deposits over extensor surfaces and severe myositis. Whole‐body magnetic resonance revealed bilateral generalized myositis and he was diagnosed with juvenile dermatomyositis. Esophagogram, cardiac and pulmonary evaluation and myositis‐specific autoantibodies were normal. He received corticosteroids, methotrexate, immunoglobulins, and hydroxychloroquine for 5 months with significant improvement. His family, however, was reluctant to maintain the therapy and returned to Romania, stopping all medications. For the next 8 months he did not receive any treatment, developing failure to thrive, worsening proximal myopathy, generalized deposition of calcium, and significant joint contractures that led to a complete loss of autonomy. He was treated at a Romanian reference center with corticosteroids, mycophenolate, immunoglobulins and IV cyclophosphamide without improvement, moving back to Spain for therapy at the age of 7. Physical examination revealed severe weakness with diffuse hardening of skin and subcutaneous tissue, nodular deposits of calcium as well as significant collections of calcium over his joints with focal ulcerations and joint contractures that interfered with all daily activities (Figure 1). Diffuse calcification was observed in X‐ray images.

**FIGURE 1:**
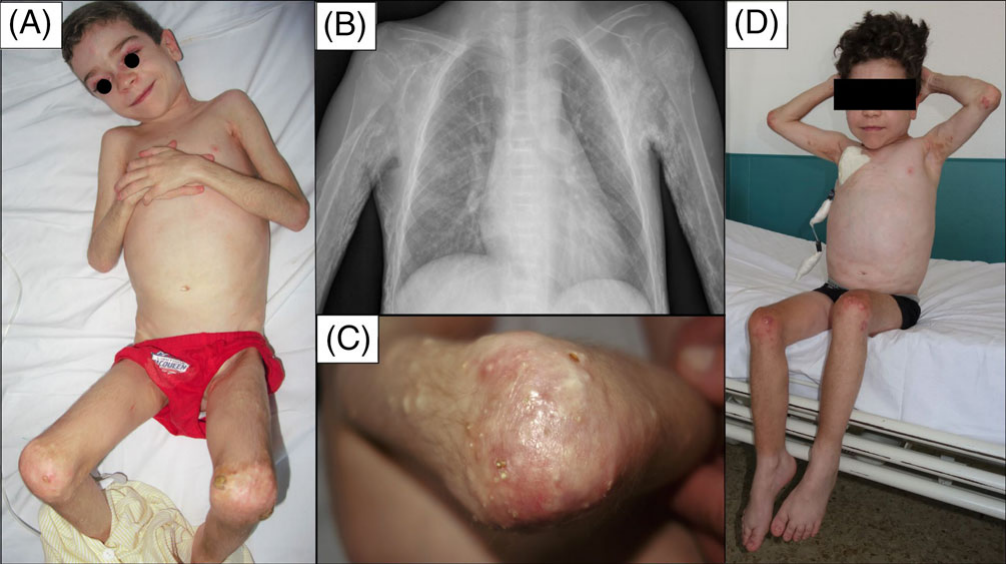
Seven‐year old boy with dermatomyositis showing heliotrope erythema, periungual telangiectasia and extensive calcium deposition with exoskeleton formation (A, C) confirmed with X‐ray imaging (B). Clinical improvement with calcium softening and improvement of articular balance (D)

He was hospitalized and systemic corticosteroids (intravenous methylprednisolone 30 mg/kg for 3 days followed by oral prednisone 60 mg/kg), immunoglobulins (2 g/kg monthly), subcutaneous methotrexate (15 mg per week), hydroxychloroquine (75 mg per day), tacrolimus (1.5 mg per day), intravenous pamidronate (1 mg/kg monthly), topical sodium metabisulfite over ulcerations and intensive physiotherapy were started. Tacrolimus was substituted by oral baricitinib (2 mg per day) after interferon signature revealed high expression of type 1 interferon and lack of improvement.

During the next months the patient's general condition, autonomy and muscle strength improved markedly, although it took 5 months to see evident clinical benefit. Creatinine kinase levels were over 600 U/L before starting baricitinib and normalized after 6 months of treatment. Calcium deposits progressively softened and articular balance increased significantly enabling him to ride his bicycle again. After 7 months, he continues with infusions of immunoglobulins and pamidronate, hydroxychloroquine, methotrexate and baricitinib with no adverse events.

Calcinosis associated with juvenile dermatomyositis (JDM) appears in up to 40% of the patients. Common complications include muscle atrophy, contractures, and ulceration. Rarely, calcinosis can progress to exoskeleton formation and is one of the most treatment‐refractory complications. Calcinosis appears 1–3 years after disease onset and indicates persistent inflammatory activity. Risk factors include treatment delay, chronic persistent disease and positive NXP2 antibodies.^1^

Treatment of calcinosis is challenging and frequently disappointing. Lack of strong evidence and difficulties regarding the evaluation of treatment response are additional pitfalls. The mainstay of treatment is to control the inflammation of the underlying dermatomyositis. If necessary, calcinosis‐specific treatment options should be considered.^2^

Increasing evidence support the benefits of JAK inhibition in the treatment of dermatomyositis, especially in patients with high expression of type‐1 interferons. Specific reports on the benefit in calcinosis are yet sparse and of uneven quality.^3^

Among the many and rather disappointing alternatives for managing calcinosis, at present bisphosphonates seem to be the best option, although improvement may have a delay of months to years.^4^ Intravenous pamidronate is the most frequently used, at one to three monthly intervals, which can be combined with immunoglobulins during hospitalization.

Topical sodium metabisulfite, a more stable derivate of thiosulphate, may be useful over superficial or ulcerated calcium deposits.^5^

In summary, severe deposits of calcium in the setting of JDM can be prevented with close follow‐up for early escalation of dermatomyositis‐specific treatment. The use of JAK inhibitors appears safe in pediatric population and its specific usefulness in dermatomyositis‐related calcinosis, as in our case, seems promising. Furthermore, calcium‐specific directed therapies, especially bisphosphonates, should also be considered. Multidisciplinary approach is mandatory and even though the outcome of these patients remains poor, positive results may appear after several months or up to years.

## AUTHOR CONTRIBUTIONS

Manuel Agud‐Dios had full access to all of the data in the study and take responsibility for the integrity of the data and the accuracy of the data analysis. Study concept and design: Manuel Agud‐Dios. Acquisition, analysis, and interpretation of data: Manuel Agud‐Dios, Jorge Arroyo‐Andres, Carmen Rubio‐Muñiz, Carlos Zarco‐Olivo, Alba Calleja‐Algarra, Sara Isabel Palencia Perez, Jaime de Inocencio. Drafting of the manuscript: Manuel Agud‐Dios, Jorge Arroyo‐Andres. Critical revision of the manuscript for important intellectual content: Sara Isabel Palencia Perez, Jaime de Inocencio. Statistical analysis: None. Obtained funding: None. Administrative, technical, or material support: Alba Calleja‐Algarra, Carlos Zarco‐Olivo. Study supervision: Carlos Zarco‐Olivo, Alba Calleja‐Algarra, Sara Isabel Palencia Perez.

## CONFLICT OF INTEREST

The authors declare no potential conflict of interest.

## WRITTEN CONSENT

Written consent for publication by both parents of the patient was obtained.

## Data Availability

The data that support the findings of this study are available from the corresponding author upon reasonable request.
